# Politics dictating on science is like a gunshot in a concert

**DOI:** 10.1177/00207640231208373

**Published:** 2023-10-27

**Authors:** Sergio Della Sala

**Affiliations:** Human Cognitive Neuroscience, Psychology, University of Edinburgh, Edinburgh, UK

**Commentary** to ‘Havana Syndrome: A Post Mortem’

By Robert E. Bartholomew and Robert W. Baloh

*International Journal of Social Psychiatry*

In their requiem to the so-called Havana syndrome, Bartholomew and Baloh list five factors that contributed to its creation. I will elaborate on one of them, namely that this novel entity was forged with unreliable data.

The concern raised by US diplomatic personnel about a variety of symptoms they were suffering from while stationed in Havana was branded ‘Sonic attack’ by some US politicians and by the press, as the diplomats heard unusual sounds at the time of their ailment. The researchers and the clinicians (Hoffer et al., 2018; Swanson et al., 2018) who examined some of these diplomats in the attempt to better understand their symptoms, accepted the assumption of such implausible cause, specifically targeting US personnel yet sparing anybody else living or working within the same premises, and framed their investigation within it. Discussing the dire clinical and political consequences of the Havana syndrome, Valdés-Sosa (2023) interpreted this practice as a confirmation bias, that is the tendency to search for information supporting prior views. Accordingly, the researchers would have fished for data to fit their conclusions to the political narrative. However, as Michel de Montaigne writing in the 16th century stated, before asking ‘How does this happen?’, one should ask ‘But does it happen?’. I maintain that the data do not constitute a new clinical entity, as such Havana syndrome does not exist and has never existed; hence, debating its cause is moot.

The main evidence for the extraordinary claim of a previously unrecognised syndrome possible due to the effect of an unknown acoustic weapon is based on the testing of six of the affected diplomats with a battery of 37 neuropsychological tests divided into 10 cognitive domains, which revealed apparent cognitive impairments in all cases. The data are reported in the supplemental material (eTable 2) of Swanson et al.’s (2018) paper and are utterly insubstantial. The authors considered as impaired any score below the 40th percentile and classed as pathological the performance of individuals scoring below this threshold on at least one of the tests. The authors reported that all six people who were tested showed impairment in at least one cognitive function, supporting their hypothesis that these people had sustained some form of neurological injury even if no sign of head trauma could be identified. Assessing any group of people with a random battery of tests using such a high threshold would result in most, if not all, of them performing below the cut-off score in one or another of the tests. Adopting the same methods for defining impairments, we ran a simulation 1000 times (Della Sala & McIntosh, 2018). In all our simulations everybody was affected; using Swanson et al.’s (2018) criteria it is very unlikely to perform normally.

In an intriguing twist, addressing these criticisms, the authors refrained from defending the singular choice of the 40th percentile threshold (Hampton et al., 2018). They argued instead that they used a different criterion from the one they had reported in the original paper, which they referred to as ‘within-individual’ deviations from the assessed individual’s ‘respective means’. This means that they considered as pathological any performance that would not align entirely with that of the other 36 scores obtained by each individual. However, without reference to proper norms, such within-individual approach would be meaningless (Binder et al., 2009). The attempted amendment was worse than the original fallacy (Della Sala et al., 2018).

Leaving aside the issue of how the data were really analysed, the first approach proposed would violate all accepted criteria to define pathology within evidence-based cognitive sciences, and the second would be methodologically senseless. It is difficult to accept that a publication like this, based on the most obvious p-hacking, could have passed a thorough reviewing process (McIntosh & Della Sala, 2018). For these reasons, at the very least JAMA should have either published an Erratum, explaining clearly and unambiguously the methods actually used in the study, or the paper should have been retracted (Cortex Editorial Board, 2018).

Moreover, the profile of good and poor performances across the six individuals originally assessed (Swanson et al., 2018) does not configure a coherent pattern, making it impossible to advance any neuropsychological diagnosis, or in fact identify a syndrome due to brain damage caused by a unique organic cause (Della Sala & Cubelli, 2018).

Following the initial reports, hundreds of people not related to the US nor working within embassies claimed to show malaise like that suffered by the US personnel in Havana. Short of considering being a US diplomat a symptom, should we not embrace a loftier view in trying to account for this phenomenon, as Bartholomew and Baloh suggest?

The anguish and debilitation lamented by all those who suffer from these symptoms, should be taken very seriously, and addressed consequently. However, rather than postulating a questionable new syndrome, it would prove advisable to conform to the prescription of parsimony (Danek et al., 2022) and analyse the data for what they can reveal. Abiding with the pressure of taming the data to the demand of politicians, granting them undue authority in their stance (Krylov, 2021), contradicts the very essence of scientific endeavour (Popper, 1994). I am not arguing the science should be naively apolitical or that data should be exempt from political interpretation (see Ball, 2021); rather I maintain that scientists should aim at collecting sound data with impeccable methods, not piloted by a priori political frames. Had we done so, the Havana syndrome would have never appeared.

## References

[bibr1-00207640231208373] Ball P. (2021). Science is political, and we must deal with it. The Journal of Physical Chemistry Letters, 12(27), 6336–6340. 34261323 10.1021/acs.jpclett.1c02017

[bibr2-00207640231208373] Binder L. M. Iverson G. L. Brooks B. L. (2009). To err is human: “Abnormal” neuropsychological scores and variability are common in healthy adults. Archives of Clinical Neuropsychology, 24(1), 31–46. 19395355 10.1093/arclin/acn001

[bibr3-00207640231208373] Cortex Editorial Board. (2018). Editorial – responsibility of neuropsychologists: The case of the “sonic attack”. Cortex, 108, A1–A2. 10.1016/j.cortex.2018.10.001 30340749

[bibr4-00207640231208373] Danek A. Rainer T. Della Sala S. (2022). Ockham’s razor, not a barber’s weapon but a writer’s tool. Brain, 145(6), 1870–1873. 35485576 10.1093/brain/awac159

[bibr5-00207640231208373] Della Sala S. Cubelli R . (2018). Alleged “sonic attack” supported by poor neuropsychology. Cortex, 103, 387–388. 29709238 10.1016/j.cortex.2018.03.006

[bibr6-00207640231208373] Della Sala S. McIntosh R. D . (2018). Cognitive impairments that everybody has. Journal of Neurology, 265(7), 1706–1707. 29845375 10.1007/s00415-018-8914-8

[bibr7-00207640231208373] Della Sala S. McIntosh R. D. Cubelli R. Kacmarski J. A. Miskey H. M. Shura R. D . (2018). Cognitive symptoms in US government personnel in Cuba: The mending is worse than the hole. Cortex, 108, 287–288. 30360897 10.1016/j.cortex.2018.10.002

[bibr8-00207640231208373] Hoffer M. Levin B. Snapp H. Buskirk J. Balaban C. (2018). Acute findings in an acquired neurosensory dysfunction. Laryngoscope Investigative Otolaryngology, 4(1), 124–131. 30828629 10.1002/lio2.231 PMC6383299

[bibr9-00207640231208373] Hampton S. Swanson R. L. Smith D. H. (2018). In reply: Neurological symptoms in US government personnel in Cuba. JAMA, 320(6), 604–605. 30120475 10.1001/jama.2018.8737 PMC9353731

[bibr10-00207640231208373] Krylov A. (2021). The perils of politicizing science. The Journal of Physical Chemistry Letters, 12, 5371. 34107688 10.1021/acs.jpclett.1c01475

[bibr11-00207640231208373] McIntosh R. D. Della Sala S. (2018). Neuropsychological impairments that everybody has. The Psychologist (BPS). https://www.bps.org.uk/psychologist/neuropsychological-impairments-everybody-has

[bibr12-00207640231208373] Popper K. R. (1994). The myth of the framework: In defence of science and rationality. Routledge.

[bibr13-00207640231208373] Swanson R. Hampton S. Green-McKenzie J. Diaz-Arrastia R. Grady M. S. Verma R. Smith D. (2018). Neurological manifestations among US government personnel reporting directional audible and sensory phenomena in Havana, Cuba. JAMA, 319(11), 1125–1133. 29450484 10.1001/jama.2018.1742 PMC5885885

[bibr14-00207640231208373] Valdés-Sosa M. J. (2023). How ‘Anomalous Health Incidents’ in Cuba Sidelined Science. Scientific American. https://www.scientificamerican.com/article/how-anomalous-health-incidents-in-cuba-sidelined-science/

